# Supplementary material for: Programmable CRISPR‐Cas transcriptional activation in bacteria
Source: Mol Syst Biol. 2020 Jul 13;16(7):e9427. doi: 10.15252/msb.20199427 (PMC7356669; doi:10.15252/msb.20199427)
Supplement: Supplementary file 1 — Appendix [file MSB-16-e9427-s001.pdf]

Appendix

Programmable CRISPR-Cas transcriptional activation in bacteria

Hsing-I Ho[1], Jennifer R. Fang[2], Jacky Cheung[3], Harris H. Wang[1,4*]

Affiliations:

[1]Department of Systems Biology, Columbia University, New York, NY, USA.

[2]Department of Biological Sciences, Columbia University, New York, NY, USA.

[3]Department of Computer Science and Biology, Columbia University, New York, NY, USA

[4]Department of Pathology and Cell Biology, Columbia University, New York, NY, USA

*Correspondence to: hw2429@columbia.edu

This PDF file includes:

Appendix Tables S1-S7
Appendix Figures S1-S9

Table of Contents

Appendix Table S1: Bacterial strains and species used in this study

Appendix Table S2: Plasmids used in this study

Appendix Table S3: dCas9 fusion candidates for CasTA

Appendix Table S4: Candidates characterized from dCas9-AsiA directed evolution

Appendix Table S5: N20 of gRNAs used in this study

Appendix Table S6: Genomic targets tested with CRISPRa

Appendix Table S7: Synthetic sequences used in this study

Appendix Figure S1: Diagram of CasTA platform

Appendix Figure S2: Optimization of selection stringency for CasTA selection platform.

Appendix Figure S3: Evaluation of different dCas9 transcription activator fusion strategies

Appendix Figure S4: Sequence profiling of AsiA variant libraries after PCR mutagenesis.

Appendix Figure S5: Characterization of dCas9-AsiA mediated CRISPRa.

Appendix Figure S6: Growth of cells expressing dCas9-AsiA.

Appendix Figure S7: Specificity of gene activation using dCas9-AsiA_m2.1.

Appendix Figure S8: Using dCas9-AsiA_m2.1 to activate genomic targets.

Appendix Figure S9: Bacterial CRISPRa screen to identify new orthogonal inducible promoters.

Appendix Table S1 Bacterial strains and species used in this study

Species	Strain name	Genotype	Note
Escherichia coli	BW25113	*F-, DE(araD-araB)567, lacZ4787(del)::rrnB-3, LAM-, rph-1, DE(rhaD-rhaB)568, hsdR514*	Wild type cell
Escherichia coli	WT-GFP	*F-, Δ(araD-araB)567, ΔlacZ4787(::rrnB-3), λ-, rph-1, Δ(rhaD-rhaB)568, hsdR514, att::[ϕ21 Wj89-GFP]*	Wild type cell chromosomally inserted with GFP cassette
Escherichia coli	JEN202	*F-, ΔrpoZ*	Deletion of omega subunit of RNAP
Salmonella enterica	Serovar Typhi Ty2		Source: ATCC 700931
Klebsiella oxytoca	M5A1		Source: DSM 7342

Appendix Table S2 Plasmids used in this study

Plasmid name	Description	Promoter for GOI	Antibiotics resistance	Replication origin
pdCas9-linker	For constructing dCas9 fusion candidate library	pTetO	Cam	p15A
pgRNA-bacteria	For constructing different gRNAs plasmids	J23119	Carb	ColE1
pWj89	Expressing GFP under weak promoter	J23117	Kan	Sc101
pWJ96	Expressing GFP under medium promoter	J23116	Kan	Sc101
pWJ97	Expressing GFP under strong promoter	J23110	Kan	Sc101
pdCas9-AsiA	Expressing dCas9 fusion AsiA	pTetO	Cam	p15A
pdCas9-AsiA_m1.1	Expressing dCas9 fusion AsiA variant 1.1	pTetO	Cam	p15A
pdCas9-AsiA_m2.1	Expressing dCas9 fusion AsiA variant 2.1	pTetO	Cam	p15A
pdCas9-AsIA-wRBS	pdCas9-AsiA with modified RBS sequnce from B0034 to B0033	pTetO	Cam	p15A
pdCas9-AsiA_m2.1-wRBS	pdCas9-AsiA_m2.1 with modified RBS sequnce from B0034 to B0033	pTetO	Cam	p15A
pHH34	Expressing Spec resistance gene under weak promoter	J23117	Kan	Sc101
pHH35	Expressing Bleo resistance gene under weak promoter	J23117	Kan	Sc101
pHH36	Expressing Kan resistance gene under weak promoter	J23117	Kan	Sc101
pHH37	Expressing KanR-ssrA under weak promoter	J23117	Kan	Sc101
pHH38	Constitutively expressed gRNA-H4 and Bleo resistance gene, serving for dual antibiotic selection	J23119 (gRNA-H4), J23117 (BleoR)	Carb	ColE1
pHH39	Expressing mScarlet-I under strong promoter and GFP under weak promoter	J23110 (mScarlet-I), J23119 (GFP)	Kan	Sc101
pHH40	Expressing dCas9-AsiA_m2.1 and gRNA-H22	pTetO (dCas9-AsiA_m2.1), J23119 (gRNA-H22)	Cam	ColE1
pHH41	Expressing dCas9-AsiA_m2.1 and gRNA-H23	pTetO (dCas9-AsiA_m2.1), J23119 (gRNA-H23)	Cam	ColE1
pHH42	Expressing dCas9-AsiA_m2.1 and gRNA-H24	pTetO (dCas9-AsiA_m2.1), J23119 (gRNA-H24)	Cam	ColE1

Appendix Table S3 dCas9 fusion candidates for CasTA

Candidate	Category	Binding partner of RNAP	Sequence	Note	References
B42	RNAP binding	Unspecified	GINKDIEECNAIIEQFIDYLRTGQEMPMEMADQAINVVPGMTPKTILHAGPPIQPDWLKSNGFHEIEADVNDTSLLLSGDAS		DOI:https://doi.org/10.1016/0092-8674(87)90015-8
BTAD1	RNAP binding	Unspecified	AEGALDLARAQDLASAAEKARSAGDLCHARDLLRRALDLWDGEVLAGVPGPYAQTQRVRLGEWRLQLLETRLDMDLDQGCHAEAVSELTALTAAHPLRERLRELLMLALYRSGRQAEALAVYADTRRLLADELGVDPRPGLQELQQRILQADPALA	Bacterial transcription activation domain from Streptomyces antibiotic regulatory protein	DOI: 10.1016/j.jmb.2007.02.096
BTAD2	RNAP binding	Unspecified	PPSTVDVNRFERDADDGQELLQRGDAAGGTKLGHALALWRGPALADVVASGRLFSYVTRLEELRFRILELRIEADLATGRHRELVSELKSLVLAHPLHEHLHGLLMLALHRSGRPHEALEVYRSVRHKMIEDLALEPAQDFATLHHTLLSDSPPEA	Bacterial transcription activation domain from Streptomyces antibiotic regulatory protein	DOI: 10.1186/1471-2180-3-3
GreA	Transcription factor	Beta and beta' subunit	MQAIPMTLRGAEKLREELDFLKSVRRPEIIAAIAEAREHGDLKENAEYHAAREQQGFCEGRIKDIEAKLSNAQVIDVTKMPNNGRVIFGATVTVLNLDSDEEQTYRIVGDDEADFKQNLISVNSPIARGLIGKEEDDVVVIKTPGGEVEFEVIKVEY	Type II transcription factor	DOI: 10.1093/emboj/cdg610
DksA	Transcription factor	Unspecified	MQEGQNRKTSSLSILAIAGVEPYQEKPGEEYMNEAQLAHFRRILEAWRNQLRDEVDRTVTHMQDEAANFPDPVDRAAQEEEFSLELRNRDRERKLIKKIEKTLKKVEDEDFGYCESCGVEIGIRRLEARPTADLCIDCKTLAEIREKQMAG	Type II transcription factor	DOI: 10.1016/j.jmb.2011.12.028
DksA D74E	Transcription factor	Unspecified	MQEGQNRKTSSLSILAIAGVEPYQEKPGEEYMNEAQLAHFRRILEAWRNQLRDEVDRTVTHMQDEAANFPDPVDRAAQEEEFSLELRNRDRERKLIKKIEKTLKKVEDEDFGYCESCGVEIGIRRLEARPTADLCIDCKTLAEIREKQMAG	DskA mutant with higher binding affinity with RNAP	DOI: 10.1016/j.jmb.2011.12.028
DksA D74N	Transcription factor	Unspecified	MQEGQNRKTSSLSILAIAGVEPYQEKPGEEYMNEAQLAHFRRILEAWRNQLRDEVDRTVTHMQDEAANFPDPVDRAAQEEEFSLELRNRDRERKLIKKIEKTLKKVEDEDFGYCESCGVEIGIRRLEARPTADLCIDCKTLAEIREKQMAG	DskA mutant with higher binding affinity with RNAP	DOI: 10.1016/j.jmb.2011.12.028

SoxS G32A	Transcription factor	Alpha subunit	MSHQKIIQDLIAWIDEHIDQPLNIDVVAKKSAYSKWYLQRMFRTVTHQTLGDYIRQRRLLLAAVELRTTERPIFDIAMDLGYVSQQTFSRVFRRQFDRTPSDYRHRL	Mutant variant with defective DNA binding ability	DOI: 10.1016/s0022-2836(02)00782-9
N4SSB	Phage protein	Beta and beta' subunit	MSNLFGNLAGQAAKAEKATDNLGGGFGAKESDIYLATLKVAYAGKAASGANFIQIIADLTDLDGHSAGEYREQLYITSGTEKGCKCTYEKNGKEYFLPGYTVINDILVMTSGETIPEAVFEEKVVNVYDFDEKKEVAKSVMVPVNAIGGKFAVAILKSEEDKQTKDGSGNYVSTGETRFTNTIEKVFHPDLHLTVVEAEELTERGKELTVEEAVFWDKWLEKNKGVTRDKTTKGGASGKAGQPPKPGATNTGAGASAAKSLFGKK	Phage protein	DOI: 10.1126/science.275.5306.1655
MotA-N	Phage protein	Sigma factor	DLGNAVVNSNIGVLIKKGLVEKSGDGLIITGEAQDIISNAATLYAQENAPELLKKRATRKAREITSDMEEDKDLMLKLLDKNGFVLKKVEIYRSNYLAILEKRTNGIRNFEINNNGNMRIFGYKMMEHHIQKFTDIGMSCKIAKNGNVYLDIKRSAENIEAVITVA	MotA with truncation of DNA binding domain at the C-terminus	DOI: 10.1128/JB.184.14.3957-3964.2002
AsiA	Phage protein	Sigma factor	MNKNIDTVREIITVASILIKFSREDIVENRANFIAFLNEIGVTHEGRKLNQNSFRKIVSELTQEDKKTLIDEFNEGFEGVYRYLEMYTNK	Phage protein	DOI: 10.1128/jb.175.1.85-93.1993
ω	RNAP subunit	Sigma factor	MARVTVQDAVEKIGNRFDLVLVAARRARQMQVGGKDPLVPEENDKTTVIALREIEEGLINNQILDVRERQEQQEQEAAELQAVTAIAEGRR		DOI: 10.1101/gad.12.5.745

Appendix Table S4 Candidates characterized from dCas9-AsiA directed evolution

Cycle	Mutations	Frequency	Note
1st	V58I, E60K, linker S1C	0.76	dCas9-AsiA_m1.1
1st	A15V,	0.04	dCas9-AsiA_m1.2
1st	linker S1C	0.04	dCas9-AsiA_m1.3
1st	E45K,	0.02	
1st	I70T, linker S1C	0.02	
1st	L84S, linker S1C	0.02	
1st	E28D	0.02	
1st	D6E, I12V, F77S	0.02	
1st	WT	0.04	
2nd	Q51R, V58I, E60K, liner S1C	0.5	dCas9-AsiA_m2.1
2nd	I40V, V58I,S59R, E60K, E85V, linker S1C	0.08	
2nd	R23H, Q51P, V58I, E60K, Y81N, linker S1C	0.08	
2nd	N29K, V58I, E60K, T88N, linker S1C	0.08	plasmid unstable
2nd	V58I, E60K, L61Q, linker S1C	0.08	
2nd	N4I, N32K, V58I, E60K, linker S1C	0.08	plasmid unstable
2nd	V58I, E60K, linker S1C	0.08	dCas9-AsiA_m1.1

Appendix Table S5 N20 of gRNAs used in this study

ID	Target	N20	Purpose
H1	WJ89	ATGTAACACCGTGCGTGTTG	Test effects of different gRNA target positions
H2	WJ89	GAAGATCCGGCCTGCAGCCA	Test effects of different gRNA target positions
H3	WJ89	GGCTCGAGTCGACAGTTCAT	Test effects of different gRNA target positions
H4	WJ89	CTACGGAACTCTTGTGCGTA	Test effects of different gRNA target positions
H5	WJ89	GCAAAAGCTCATTTCTGAAG	Test effects of different gRNA target positions
H6	WJ89	AACTCTTGTGCGTA	Test shorter seed sequence
H7	WJ89-GFP	TTGACAGCTAGCTCAGTCCT	Use dCas-AsiA_m2.1 for CRISPRi
H8	WJ89-GFP	GCTAGCGAATTCCTTTAAAG	Use dCas-AsiA_m2.1 for CRISPRi
H9	WJ89-GFP	CCATCTAATTCAACAAGAAT	Use dCas-AsiA_m2.1 for CRISPRi
H10	WJ89-GFP	GAATTAGATGGTGATGTTAA	Use dCas-AsiA_m2.1 for CRISPRi
H11	mScarlet-I	TCTGGGTGCCTTCATACGGA	CRISPRi for mScarlet
H13	*cadB*	TTTATGTAATAAAAATTATG	Test CRISPRa on genomic targets
H15	*zraP*	GCTGTCAGAAAGGGATGAGC	Test CRISPRa on genomic targets
H19	*iraM*	TGCCAATTTGCTAAACATTA	Test CRISPRa on genomic targets
H20	*iraD*	ATAATACATGGCTGATTATG	Test CRISPRa on genomic targets
H21	*ycgZ*	TTTTTATCAATGTAAAGAAA	Test CRISPRa on genomic targets
H22	RS7003 promoter library	AATAATGGTTTCTTAGACGT	Induce expression of metagenomic promoter library
H23	RS7003 promoter library	AAAAGGGAATAAGGGCGACA	Induce expression of metagenomic promoter library
H24	genomic control	AAGCTGAAGAAAAATGAGCA	genomic target control for the inducible library screening
H25	dxs	CAATTTAATGATAAACTTCA	Test CRISPRa on genomic targets
H26	ffh	AGTCTTGCGCTGATTGTTCC	Test CRISPRa on genomic targets
H27	yehA	ATACCGATCAGCGCAAGCCA	Test CRISPRa on genomic targets
H28	ydiN	TTTTTACTGGCACTGTTTAT	Test CRISPRa on genomic targets
H29	idi	CTGATAAAGATTTAAAAGTC	Test CRISPRa on genomic targets
H30	WJ89	CGGTGTTACATTAGGCATAC	Test effects of different gRNA target positions
H31	WJ89	AACACGCACGGTGTTACATT	Test effects of different gRNA target positions
H32	WJ89	CGTGCGTGTTGTGGAAGATC	Test effects of different gRNA target positions
H33	WJ89	CGGATCTTCCACAACACGCA	Test effects of different gRNA target positions
H34	WJ89	GCCAAGGTGATAATCCATAG	Test effects of different gRNA target positions
H35	WJ89	TTATCACCTTGGCTGCAGGC	Test effects of different gRNA target positions
H36	WJ89	TGGATTATCACCTTGGCTGC	Test effects of different gRNA target positions
H37	WJ89	GCCTCTATGGATTATCACCT	Test effects of different gRNA target positions
H38	WJ89	ACTGTCGACTCGAGCCTCTA	Test effects of different gRNA target positions
H39	WJ89	CAGTTCATAGGTGATTGCTC	Test effects of different gRNA target positions
H40	WJ89	CTCAGGACATTTCTGTTAGA	Test effects of different gRNA target positions
H41	WJ89	CTTGTGCGTAAGGAAAAGTA	Test effects of different gRNA target positions

H42	WJ89	AACACAAACTTGAACAGCTA	Test effects of different gRNA target positions
H43	WJ89	TTTCTGAAGAGGACTTGTTG	Test effects of different gRNA target positions

Appendix Table S6 Genomic targets tested with CRISPRa

Gene ID	Gene name	Genomic location	Forward primer	Reverse primer
945729	*iraM*	1211680-1212003	ATTTCTCCCTCCTGGCAGTA	TGGAGGACACTCTTGACTGC
948851	*iraD*	4556993-4557385	AACCCGAGCGACAAACATCT	GAGTGTGGCAGTACGCTTCT
945885	*ycgZ*	1215789-1216025	CTCAGCAGGAAACTCTCGGG	CTGTTCCTCTTCCCCAGTCG
948654	*cadB*	4358697-4360031	CGGGTATCGCCTGTATTGCT	CAAACCAATGCCAGCCAACA
948507	*zraP*	4201263-4201688	GACAGCGTGGCAGAAAATCC	CTTTGGCGACCGCGTTAATT
945060	*dxs*	438315-440177	AAGGCCCGCAGTTCCTGCAT	GGCAAACCGCCGCTACTTTTC
947102	*ffh*	2746434-2747795	CTGCAAGGTGCCGGTAAAAC	TCAAGCTGTTTGATTGCCGC
946642	*yehA*	2187380-2188414	TGGCAAGTCATGGGATGCAT	AATCGTCCGGTTTGCAGGTT
946198	*ydiN*	1772512-1773777	TTCCTGCACGGCATTAGTGT	ATCAATCGCCCCAAACCGAT
949020	*idi*	3033065-3033613	ATCTCGCGTTCTCCAGTTGG	GATCACTGCGTCTTCGTTGC
948332	*rrsA*	4035531-4037072	CTCTTGCCATCGGATGTGCCCA	CCAGTGTGGCTGGTCATCCTCTCA

Appendix Table S7 Synthetic sequences used in this study

	Sequence	Purpose	Note
Linker	SAGGGGSGGGGS	Making dCas9-TA fusion	
MS2 hairpin	GCGCACATGAGGATCACCCATGTGCT	Constructing SAM CRISPRa	
MCP-AsiA	MASNFTQFVLVDNGGTGDVTVAPSNFANGVAEWISSNSRSQAYKVTCSVRQSSAQKRKYTIKVEVPKVATQTVGGVELPVAAWRSYLNMELTIPIFATNSDCELIVKAMQGLLKDGNPIPSAIAANSGIYSAGGGGSGGGGSGGGGSMNKNIDTVREIITVASILIKFSREDIVENRANFIAFLNEIGVTHEGRKLNQNSFRKIVSELTQEDKKTLIDEFNEGFEGVYRYLEMYTNK	Constructing SAM CRISPRa	
Degradation tag	AANDENYALAA	Constructing more stringent antibiotic reporter genes	BBa_M0050
Weak RBS	TCACACAGGAC	Modulating dCas9-AsiA expression	BBa_B0033
Strong RBS	AAAGAGGAGAAA	Modulating dCas9-AsiA expression	BBa_B0034
Constitutive promoter	GTATACTTTTTTTAAAGAAAAGATTTACAAGCGCACTTTTCTTTAATATCTTACAATAATGTAAGTTTGAACAGGAGAATGTAAGCCAAAGCGATGGCTACGCATTCTCTTTCTTTGTTATACTAACACCATATTCGAGGTAGAAAATTATTTAGGAGGATAGAT	Spiked-in control for normalizing expression between samples in the screening for CRISPRa inducible promoters	Barcode (CACATGCAGTTG)

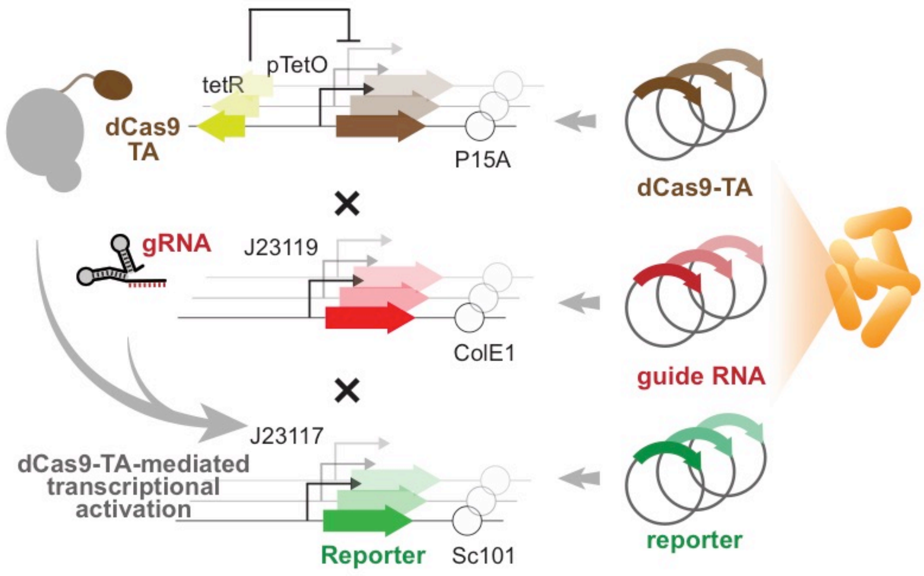

Appendix Figure S1. Diagram of CasTA platform The design of separating 3 key components of CRISPRa, dCas9-TA, gRNA, and reporter, into 3 compatible plasmids that could function in the same cell.

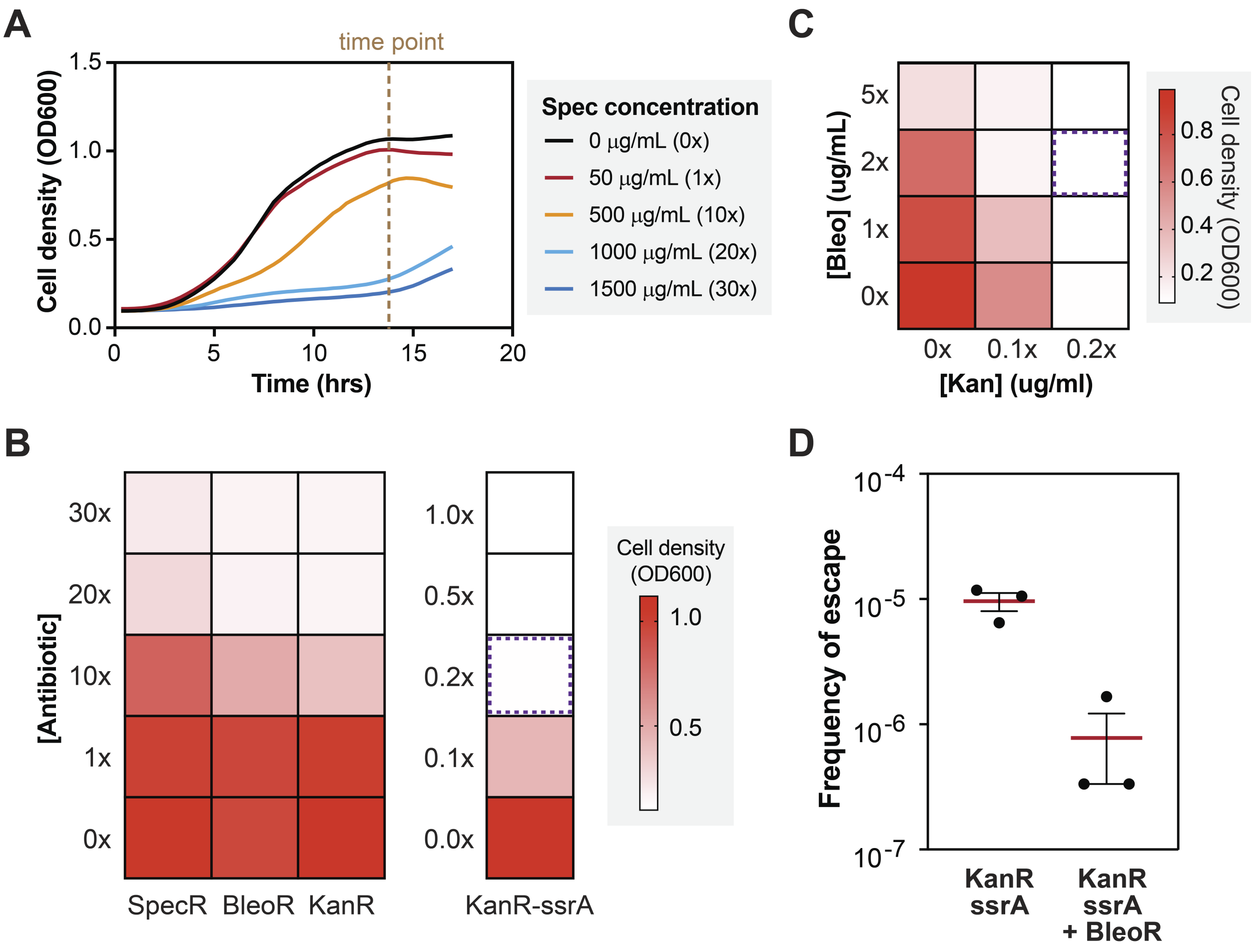

Appendix Figure S2. Optimization of selection stringency for CasTA selection platform. (A) Growth curves of *E. coli* containing pHH34 on LB media supplemented with different spectinomycin concentrations. Dotted line indicates growth phase when cell density was measured in other panels. **(B)** Selection stringency of different antibiotics using corresponding resistance genes as selection reporters (pHH34-37). KanR-ssrA: Kan resistance gene (KanR) with degradation tag (AANDENYALAA). Heat map corresponds to cell density after 14 hrs. Purple dotted outline corresponds to the antibiotic concentration used for sufficiently stringent selection. For SpecR, 1x Spectinomycin = 50 μg/ml. For BleoR, 1x Bleocin = 5 μg/ml. For KanR, 1x Kanamycin = 50 μg/ml. **(C)** Selection stringency of KanR-ssrA (x-axis) and BleoR (y-axis) dual reporter with double antibiotic selection of Kanamycin (Kan) and Bleocin (Bleo). Purple dotted outline corresponds to the antibiotic concentration used for sufficiently stringent selection. **(D)** Escape rates of using KanR-ssrA alone or KanR-ssrA and BleoR as selection reporters. Data are 3 biological replicates in each experiment. Errorbars are S.E.M.

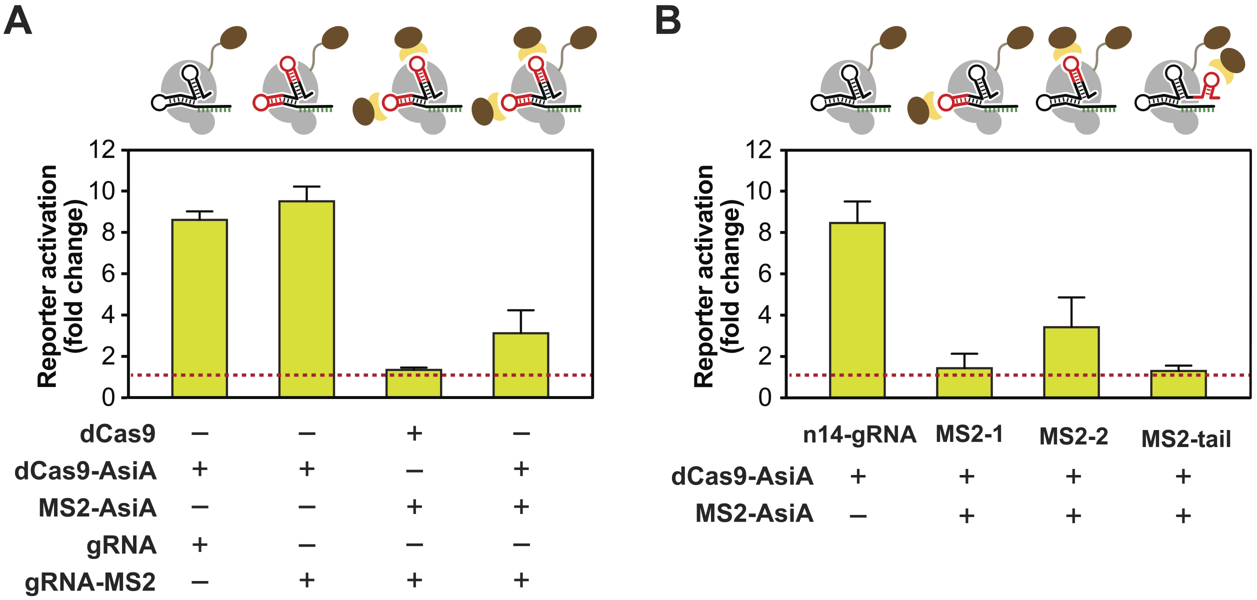

Appendix Figure S3. Evaluation of different dCas9 transcription activator fusion strategies. (A) dCas9 SAM system with modified gRNA and MS2-AsiA does not enhance CRISPRa activity. dCas9 tethered AsiA is required for facilitating gene activation. **(B)** Examination of different gRNA designs for improving CRISPRa. n14-gRNA represents design with only 14 nucleotides of the N20 seed sequence; MS2-1: incorporating MS2 hairpin structure in the first loop of the wild-type gRNA structure; MS2-2: incorporating MS2 in the second loop of the wild-type gRNA structure; MS2-tail: MS2 was fused at the 3' end of the gRNA structure. Bars are mean of 3-5 biological replicates with errorbars as S.E.M.

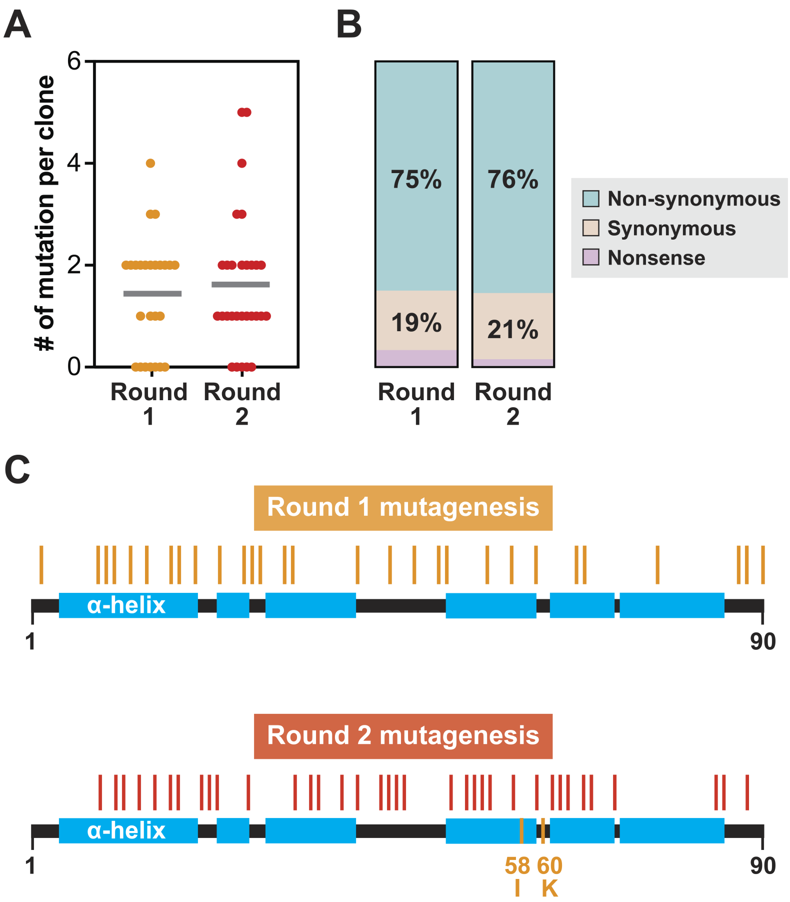

Appendix Figure S4. Sequence profiling of AsiA variant libraries after PCR mutagenesis. Sanger sequencing of AsiA variants after 2 rounds of mutagenesis **(A)** the number of mutations per variant, **(B)** the types of mutations in the protein sequences, and **(C)** mutated positions along the protein secondary structure of AsiA (indicated by colored ticks). Profiles are generated based on at least 25 randomly selected variants from each round of mutagenesis.

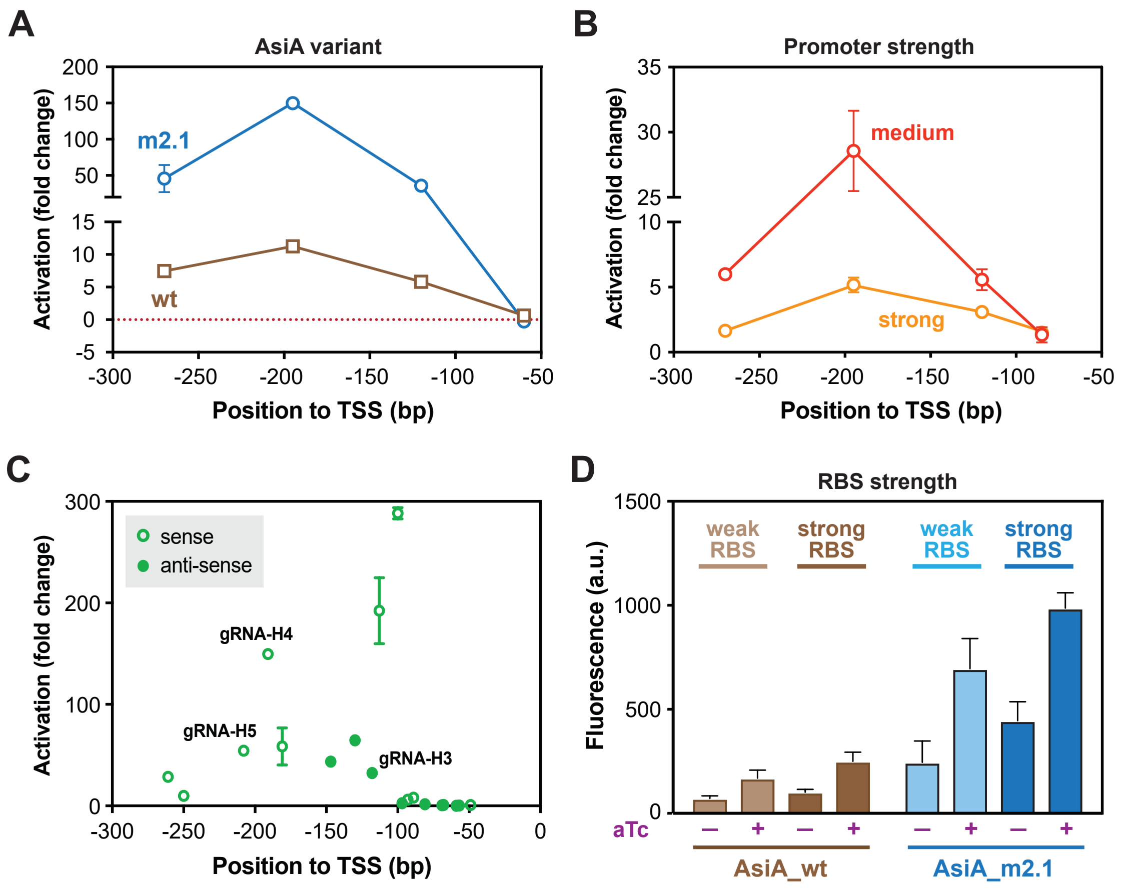

Appendix Figure S5. Characterization of dCas9-AsiA mediated CRISPRa. (A) Transcriptional activation of a weak promoter (J23117) of a GFP reporter using different gRNAs with dCas9-AsiA wild-type (wt) or mutant (m2.1). dCas9-AsiA_m2.1 (blue circles) has similar optimal gRNA targeting distance (~-190bp from TSS) as dCas9-AsiA_wt (brown squares). **(B)** Transcriptional activation using dCas9-AsiA_m2.1 with different gRNAs against a medium basal strength promoter (J23116; red circles) or strong basal strength promoter (J23110; orange circles). Induction range is found to be higher for the medium promoter than the strong promoter due to saturating absolute induction level for both promoters. **(C)** Different gRNAs targeting all NGG sites across the weak promoter (J23117) were paired with dCas9-AsiA_m2.1 to profile the optimal gRNA binding distance. The same gRNAs (H3 to H5) as used in Fig. 1 were labeled. **(D)** Increasing ribosomal binding site (RBS) strength (**Table S9**) with and without transcriptional induction (+/- aTc) of dCas9-AsiA wild-type (wt) or mutant (m2.1) generally increased the fluorescence signal of the reporter gene. Weak RBS (BBa_B0033), strong RBS (BBa_B0034). Mean from three biological replicates are plotted with errorbars as +/- S.E.M.

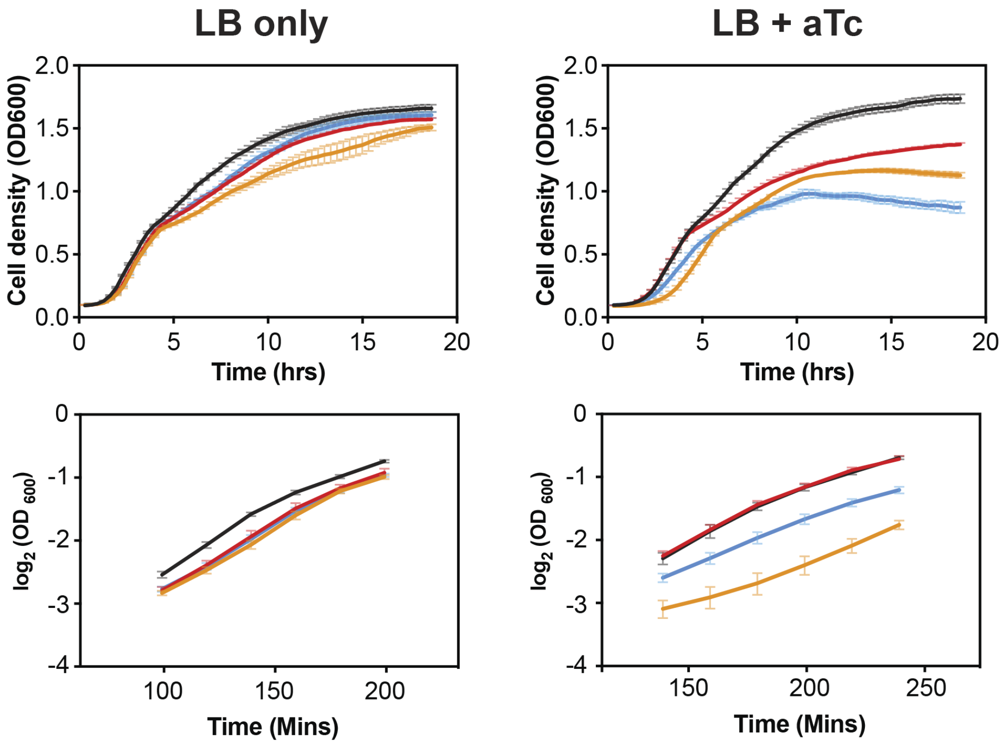

	doubling time (mins)	
	LB only	LB + aTc
BW25113 (WT)	57-68	62-77
dCas9	50-64	63-81
dCas9-AsiA	51-60	62-77
dCas9-AsiA_m2.1	53-62	65-85

Appendix Figure S6. Growth of cells expressing dCas9-AsiA. Cells carrying different dCas9-AsiA plasmids were grown in rich media with (LB+aTc) or without (LB only) dCas9 overexpression. Growth curve and doubling times in the exponential growth phase are shown. Data are three biological replicates with errorbars as +/- S.E.M.

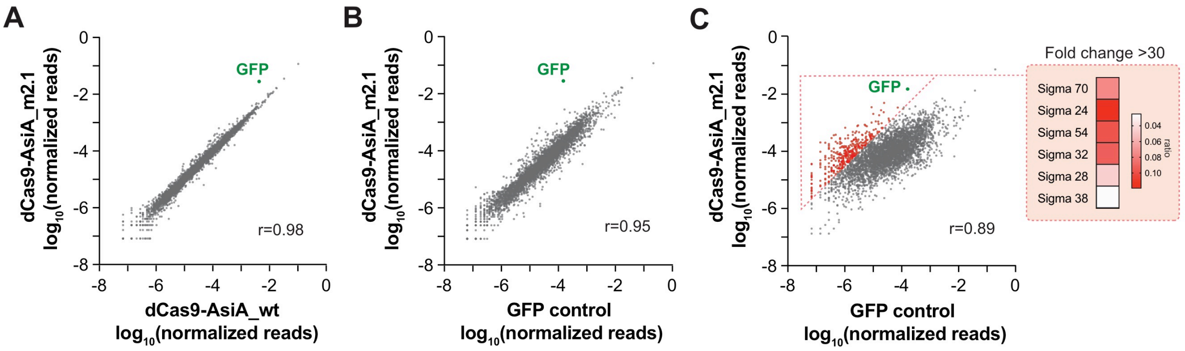

Appendix Figure S7. Specificity of gene activation using dCas9-AsiA_m2.1. (A) Transcriptomic profile of cells expressing dCas9-AsiA_wt using an optimal gRNA (gRNA-H4) targeting a GFP reporter gene on pWJ89 (x-axis) versus cells expressing dCas9-AsiA_m2.1 and the same gRNA (y-axis). **(B)** Transcriptomic profile of parental GFP control (pWJ89) cells (x-axis) versus cells expressing dCas9-AsiA_m2.1 under basal level (no ATC induction) and gRNA-H4 (y-axis). **(C)** Transcriptomic profile of parental GFP control cells (x-axis) versus with cells overexpessing dCas9-AsiA_m2.1 (with ATC induction) and gRNA-H4 (y-axis). Genes with more than 30 fold up-regulation under dCas9-AsiA_m2.1 over-expression are highlighted in red and grouped by their annotated sigma factors. Heatmap on the right indicates the ratios of highly activated (fold change >30) promoters within each group of promoters mediated by different sigma factors. r represents Pearson correlation coefficient. Each data point represents a single biological sample from the RNA-seq experiment.

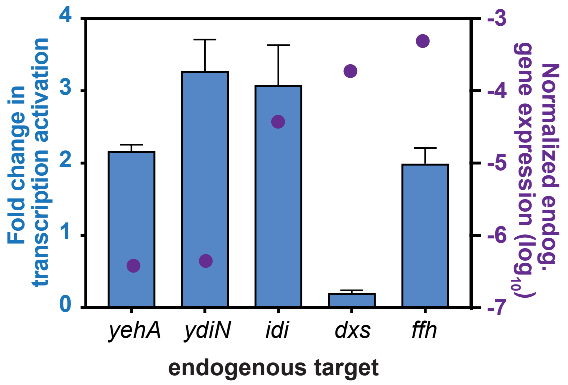

Appendix Figure S8. Using dCas9-AsiA_m2.1 to activate genomic targets. Chromosomal genes were selected to test with CasTA2.1 on gene activation. Expression was quantified using RT-qPCR, and genes with modest or no activation (<5 fold) were plotted with bars showing the activation fold change and dots showing basal expression of each gene. Data were mean of 3 biological replicas +/- SEM.

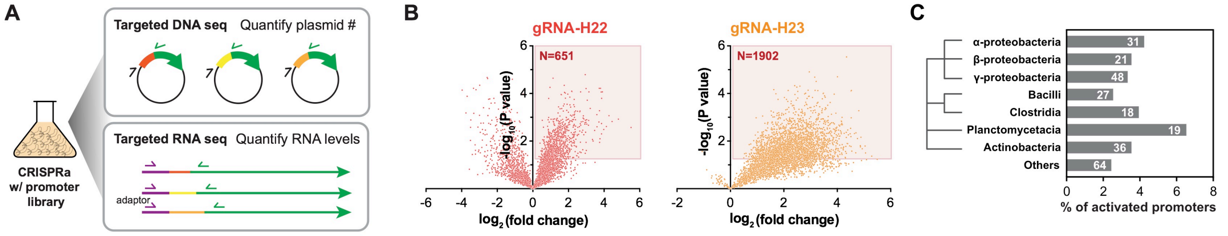

Appendix Figure S9. Bacterial CRISPRa screen to identify new orthogonal inducible promoters. (A) Using CRISPRa on a metagenomic promoter library (RS7003) to mine CasTA-inducible promoters using targeted DNAseq and targeted RNAseq. **(B)** Volcano plots of CasTA-mediated activation using two different gRNAs (gRNA-H22 and gRNA-H23) of the same promoter library, with each point in the plot corresponding to a unique promoter. Significantly activated promoters ($p<0.05$) are highlighted with the red rectangle, and the numbers of activated promoters are indicated. Data are calculated from 4 biological replicas. **(C)** The percentage of highly activated promoters (fold change >10) among all promoters of each bacterial genus. Numbers in the bars indicate the number of highly activated promoters. Dendrogram represents the phylogenic distance between each group.
